# Molecular regulation of arteriovenous endothelial cell specification

**DOI:** 10.12688/f1000research.16701.1

**Published:** 2019-07-29

**Authors:** Jennifer S. Fang, Karen K. Hirschi

**Affiliations:** 1 Department of Molecular Biology and Biochemistry, School of Biological Sciences, University of California, Irvine, Irvine, CA, 92697, USA; 2 2Departments of Medicine, Genetics, and Biomedical Engineering, Yale Cardiovascular Research Center, Yale Stem Cell Center, Yale University School of Medicine, 333 Cedar Street, New Haven, CT, 06520, USA

**Keywords:** artery, vein, endothelial cell specification, vascular development

## Abstract

The systemic circulation depends upon a highly organized, hierarchal blood vascular network that requires the successful specification of arterial and venous endothelial cells during development. This process is driven by a cascade of signaling events (including Hedgehog, vascular endothelial growth factor (VEGF), Notch, connexin (Cx), transforming growth factor-beta (TGF- β), and COUP transcription factor 2 (COUP-TFII)) to influence endothelial cell cycle status and expression of arterial or venous genes and is further regulated by hemodynamic flow. Failure of endothelial cells to properly undergo arteriovenous specification may contribute to vascular malformation and dysfunction, such as in hereditary hemorrhagic telangiectasia (HHT) and capillary malformation-arteriovenous malformation (CM-AVM) where abnormal vessel structures, such as large shunts lacking clear arteriovenous identity and function, form and compromise peripheral blood flow. This review provides an overview of recent findings in the field of arteriovenous specification and highlights key regulators of this process.

## Introduction

Systemic blood circulation depends upon a highly organized vessel network to efficiently deliver nutrient-rich blood to, and remove waste from, peripheral tissues. The mechanisms that drive development of the blood vasculature are of significant scientific interest with regard to improving our basic understanding of developmental vascular biology and to advancing the fields of personalized and regenerative medicine, where the ability to engineer blood vessels in a laboratory setting would be of significant therapeutic value. In particular, the cell signaling programs that govern the acquisition of specialized endothelial cell identities such as arteries and veins—both crucial for the formation of a functional circulatory system—are the focus of this review.

During early embryonic development, the vasculature forms when primitive endothelial cells coalesce into a primordial microvascular network. Subsequent remodeling of this primitive vasculature organizes the network into the hierarchal architecture typical of mature vessel beds. Specifically, vascular remodeling processes (including endothelial cell proliferation and migration as well as mural cell recruitment and differentiation) drive the formation of anatomically distinct large vascular structures such as arteries and veins (reviewed by dela Paz and D’Amore ^1^ in 2009). The arterial and venous sides of the systemic circulation are connected to one another at one end by the heart, and at the other end by dense networks of microvessels in the periphery; altogether, this systemic circuit supports circulation of blood throughout the body. Arteries and smaller arterioles are located upstream of the microvasculature and bear a characteristically thick layer of circumferentially organized smooth muscle cells that regulate vessel diameter to influence luminal blood flow and downstream perfusion. In contrast, the smooth muscle layer of veins and venules is thinner and less organized, resulting in a less rigid vessel wall capable of supporting the large blood volume typical of the venous side of the vasculature, and venous valves prevent luminal backflow through these vessels. Microvascular capillaries are a network of thinly walled, small-caliber blood vessels surrounded by a perivascular layer of pericytes that together support exchange of oxygen, fluid, nutrients, and waste into and out of surrounding tissue.

Throughout the vasculature, an inner endothelial layer forms the interface between the vessel lumen and the vessel wall. However, endothelial cells are not a homogeneous cell population: rather, endothelial cells of specialized vascular structures (that is, arteries, veins, and microvessels) express distinct molecular signatures ^2^ that reflect their individual location and function in the vascular tree. Moreover, acquisition of specialized endothelial cell signatures occurs prior to network formation ^1, 3^, suggesting that molecular specification of endothelial cells toward arterial or venous identities (termed arteriovenous specification) or other endothelial cell lineages (such as lymphatic or hemogenic) can drive the morphological reorganization of nascent vessel networks. Defects in vascular specification, proliferation, and remodeling can result in arteriovenous malformations (AVMs) that express both arterial and venous markers ^4^ and may even express markers of lymphatic vasculature ^5^. Collectively, these data emphasize the importance of proper endothelial cell specification for normal development and function of the vascular system.

## Early embryonic vessel development and endothelial specification

During embryogenesis, vascular endothelial cells originate from mesoderm-derived angioblasts (that is, endothelial progenitor cells) that, in mice, first appear at embryonic day 7.0 to 7.5 in the extra-embryonic yolk sac. During the process of vasculogenesis, precursor cells progressively acquire markers of endothelial cell phenotype—vascular endothelial growth factor receptor type 2 (VEGFR2), vascular endothelial cadherin, and so on ^6, 7^ —and form a primitive vascular plexus in the yolk sac and in the embryo proper ^8^. Recently, Plein *et al*. ^9^ reported that a subset of yolk sac endothelial cells that acquire hemogenic potential to become erythro-myeloid progenitor cells are also capable of re-differentiating into endothelial cells and integrating back into the yolk sac and embryonic vasculature ^9, 10^. The primitive vasculature that is comprised of these two endothelial cell sources undergoes stepwise remodeling to produce the earliest extra- and intra-embryonic vessels, which collectively form the embryo’s first closed circulatory loop ^8^. As endothelial cells of the dorsal aorta and the cardinal vein within the embryo progressively acquire expression of arterial and venous markers, respectively, the remaining endothelial cells of primitive plexi undergo sprouting angiogenesis to expand the vascular network, followed by additional remodeling and specification to further reorganize the network into a hierarchal branching architecture.

Arteriovenous specification of the endothelial cells that form the dorsal aorta and cardinal vein appears to be molecularly determined prior to the onset of systemic blood flow. Discrete, non-overlapping expression of neuropilin-1 (Nrp1) and neuropilin-2 (Nrp2) is observed in vascular plexi of chick embryos and these endothelial cell populations subsequently segregate into the earliest embryonic arteries and veins, respectively ^3^. Expression of ephrinB2, enriched in some arterial endothelial cells, and the receptor EphB4, enriched in some venous cells, is also observed in the primitive vasculature prior to the onset of blood flow ^11^. Despite these findings, the early morphogen or morphogens that first induce formation of the initial vascular plexus and support arteriovenous specification therein remain unclear. Furthermore, it is still uncertain whether these arteriovenous specification pathways are common across all vertebrate species or even whether, within the same organism, all endothelial cells synchronously acquire their arteriovenous identity via the same initiating signal(s) or downstream mechanism(s).

## Role of shear stress

The observations that embryonic arterial and venous markers are expressed prior to the onset of blood flow ^3, 11, 12^ and that blood flow is dispensable for early arteriovenous specification events in the chick embryo ^13^ suggest that the initiating arteriovenous specification event for angioblasts is not necessarily blood flow. However, blood flow is nonetheless crucial for certain arteriovenous specification events and is necessary for the maintenance of arterial identity.

Supporting this point, loss of systemic blood flow in chick ^13, 14^ and mice ^15, 16^ produces defects in arteriovenous specification and induces AVMs. For example, in Ncx1 ^−/−^ mouse embryos (which lack a heartbeat), blood flow is not required for initial formation of the dorsal aorta and the cardinal vein but is necessary to induce separation of these vessel structures and to maintain arterial marker expression and suppress venous identity genes in the dorsal aorta ^15^. Thus, early endothelial cell fate acquisition is dynamic, and hemodynamic signaling is needed to sustain arteriovenous identity in the remodeling vasculature.

Consistent with this model, expression of arterial identity genes is induced in cultured endothelial cells and is greatest when cells are exposed to shear stress magnitudes typical of arterial vessels (~15 dynes/cm ^2^), relative to higher or lower shear magnitudes ^17^. Furthermore, maintenance of arterial gene expression in cultured arterial endothelial cells requires pulsatile, not constant, flow ^14^. These data indicate that endothelial cell specification is tightly calibrated to hemodynamic flow profile and suggest that other endothelial cell types, such as venous and lymphatic, may be similarly promoted by vessel-specific flow profiles. Consistent with the idea that endothelial cell identity is plastic and influenced by hemodynamic flow, several studies show that vessel grafts generally lose markers of their vessels of origin and assume the molecular identity of their grafted location ^18^. Although the particular mechanosensitive pathways that govern these flow-sensitive specification events remain unclear, activation of mechanosensitive receptors, such as activin receptor-like kinase 1 ( *ACVRL1*, or Alk1) and Notch1 or Notch4, likely leads to the downstream transactivation of fundamental regulators of endothelial cell specification and vascular remodeling, which is discussed in greater detail below.

## Role of cell cycle control

A growing body of evidence suggests that endothelial cell cycle arrest is necessary to enable the acquisition of specialized endothelial cell phenotypes. Cell cycle control critically regulates cell fate decisions during embryonic stem cell differentiation ^19, 20^, suggesting that a similar process may occur for acquisition of specialized cell phenotypes in other contexts, such as for endothelial cells in the vasculature. In undifferentiated stem cells, cell cycle progression is tightly regulated ^21^, and cell cycle length governs both pluripotency ^20^ and cell differentiation ^19^. In blood vessels, molecular regulation of cell cycle state may similarly be required to achieve a balance between expansion and maturation of vessel networks.

Consistent with this hypothesis, angiogenic endothelial cells are highly proliferative ^22^, whereas proliferation is substantially suppressed in remodeling vessel networks undergoing arteriovenous specification ^17, 23^, particularly in developing arterial-associated vascular beds ^17^. In addition, fluid shear stress at physiologically arterial levels significantly reduces proliferation of endothelial cells in culture ^24^ and endothelial cells in mature arteries are characteristically quiescent ^25^. Recent studies show that pharmacological induction of G _1_ arrest is sufficient to enable the expression of arterial identity genes in endothelial cells in culture even in the absence of other conventional activators of arterial specification ^17^. In addition, during coronary vascular development, transition from venous to arterial endothelial cell phenotypes is associated with G _1_ growth arrest that is prevented by expression of the venous identity regulator COUP-TFII ^26^, and endothelial cell G _1_ arrest is also required for hemogenic specification ^27^. Thus, signaling pathways, including those reviewed below, may regulate endothelial specification, at least in part, by modulating cell cycle state to enable subsequent endothelial cell specification events. Whether a specific cell cycle state is necessary to enable venous and lymphatic endothelial cell specification is unclear and this is under investigation.

## Regulators of arteriovenous specification

Vascular network morphogenesis and endothelial cell specification require coordinated cell–cell signaling between endothelial cells, mural cells, and adjacent cell types. Specification of endothelial cell identity is regulated by the integrated balance of multiple cell–cell signaling pathways that antagonistically induce arterial or venous identity. In particular, an “arterialization” cascade involving Hedgehog, vascular endothelial growth factor (VEGF), Notch, and connexin (Cx) signaling plays an important role in inducing arterial specification. There is also cross-talk of this pathway with transforming growth factor-beta (TGF-β) signaling and this pathway is inhibited by regulators of venous identity, such as COUP-TFII.

### Hedgehog

Binding of the morphogen Sonic Hedgehog (Shh) to its cell surface receptor Patched-1 ( *PTCH1*) alleviates repression of the central downstream Shh effector, Smoothened (Smo). In turn, Smo induces the expression of numerous gene targets essential for embryonic development ^28^. In endothelial cells, Shh activates endothelial cell survival and alters cytoskeletal arrangement in culture ^29^, and studies in zebrafish show that Shh signaling is necessary for arteriovenous specification. Specifically, in zebrafish mutants lacking Shh, endothelial cells of the dorsal aorta fail to acquire expression of the arterial-enriched gene ephrinB2. This is thought to be the result of loss of VEGF expression in Shh-deficient somites, leading to reduced VEGF signaling and reduced downstream Notch ^30^. However, Shh also regulates endothelial cell identity independent of its stimulation of VEGF/Notch signaling. Specifically, Hedgehog represses venous identity ^31^ and promotes arterial specification via calcitonin receptor-like receptor (Crlr) signaling ^32^ as well as by directly upregulating expression of Notch signaling effectors ^33^.

### Vascular endothelial growth factor

VEGF functions at multiple levels during vasculogenesis and vessel remodeling, including during arteriovenous specification. Although loss of even a single allele of VEGF-A is sufficient to disrupt vessel formation resulting in embryonic lethality in mice ^34^, VEGF-A knockdown in zebrafish morphants preserves embryonic survival albeit with arteriovenous specification defects ^30^. Recent studies indicate that whereas early VEGF signaling governs endothelial cell development from angioblasts, mid-somitogenic VEGF signaling primarily influences arterial specification by activating Etv2, a member of the Ets family of transcription factors ^35^, to regulate downstream Notch signaling effector expression ^36, 37^.

Recent findings indicate that, in addition to its effects on Etv2 and downstream Notch, VEGF/VEGFR2 activation directly regulates the balance of signaling through either phosphatidylinositol-3-kinase (PI3K) or mitogen-activated protein kinase (MAPK) pathways to determine arterial and venous cell fates. In a small-molecule screen, Hong *et al*. ^38^ report that inhibition of PI3K signaling induces ERK1/2 (MAPK signaling) activation—a signaling pathway that regulates endothelial cell proliferation (among other functions) ^39^—to promote arterial specification. This effect is capable of rescuing arteriovenous defects of the *gridlock* zebrafish mutant, where the Notch-targeted transcription factor Hey2 is affected, indicating that MAPK signaling influences arterial identity downstream of Notch signaling ^38^. In contrast, small-molecule inhibitors of MAPK, or constitutive activation of PI3K signaling via induction of protein kinase B (Akt), prevents arterial specification and instead induces venous identity ^38^, indicating that the antagonistic relationship between MAPK and PI3K signaling pathways strongly influences endothelial cell fate.

### Notch

Members of the Notch family of transmembrane receptors, as well as their membrane-bound ligands, are expressed in multiple cell types of the developing and mature vasculature ^40^. In response to VEGF-activated expression of members of the Sox family of transcription factors (for example, Sox7, Sox17, and Sox18) ^41^ or stimulation of the Wingless/Integrated (Wnt) signaling pathway ^42^, primordial endothelial cells are induced to express the transmembrane receptor Notch1 and its ligand Dll4 ^43^. Notch1 and related endothelial-expressed Notch receptors are activated by membrane-bound Notch ligands of adjacent endothelial cells (homocellular signaling) as well as those expressed by other stromal cell types (heterocellular signaling). Indeed, the developing vasculature expresses multiple Notch ligand and receptor types, and some are restricted to specific regions of the expanding and remodeling vascular tree ^40^. Binding of ligand to the Notch receptor results in proteolytic cleavage of the Notch intracellular domain, which translocates to the nucleus and binds to and activates DNA-binding protein RBPJκ, resulting in transcription of genes that influence endothelial cell cycle status ^17^ and function, leading to induced expression of arterial identity genes ^44^.

In animals treated with Notch inhibitors or in transgenic animals lacking either Notch ligands or receptors, sprouting angiogenesis and arteriovenous specification fail to occur normally ^17, 22, 45– 47^. Instead, vascular endothelial cells hyperproliferate and do not properly remodel into arteriovenous networks ^17, 45^. In *gridlock* zebrafish mutants, ephrinB2 expression is lost and formation of the dorsal aorta is compromised but the cardinal vein is enlarged ^12^.

One possible explanation for the central role for Notch in arteriovenous specification is as a mechanism to couple mechanosensory receptor signaling to downstream endothelial cell specification pathways. Fluid shear stress activates Notch signaling in endothelial cells in a dose-dependent fashion with Notch activation peaking at ^17^ or slightly above ^48^ physiologically arterial levels of shear. Ablation of Notch1 signaling compromises classic flow-sensitive endothelial cell responses, including quiescence ^17^ and cell alignment ^48^, whereas constitutive activation of Notch4 induces focal vessel enlargement by disrupting normal hemodynamic signaling ^49^. Although the exact mechanosensory signaling complex or complexes that render Notch signaling flow-sensitive have yet to be identified, ligand-dependent Notch activation is force-dependent ^50, 51^, which suggests that the Notch receptor itself may participate in an as-yet-undescribed mechanosensory complex.

Other studies suggest that Notch may also enable arteriovenous specification by determining the cell cycle state of remodeling endothelial cells. In response to flow, Notch signaling activation alters the expression of cell cycle regulators ^17^. In addition, Notch-mediated G _1_ arrest is required for acquisition of arterial ^17, 45^ as well as hemogenic ^27^ cell fates. In contrast, suppression of Notch signaling by COUP-TFII drives venous specification ^52^, and transgenic ablation of Notch signaling components enhances lymphatic endothelial cell specification ^53^. Taken together, these data suggest that, in the developing vasculature, Notch signaling may play a central role in precisely coupling endothelial cell cycle state to hemodynamic flow sensing to achieve proper fate specification. However, it is still unclear whether venous and lymphatic endothelial cell fates are similarly specified in distinct cell cycle states, which requires further intensive investigation. Nonetheless, in support of this hypothesis, dysregulated Notch signaling leads to focal appearance of AVMs at sites of high flow that are associated with failure to acquire (or maintain) specialized endothelial cell identities ^49, 54^. Whether in animals lacking Notch (or Alk, see below) signaling AVMs are a direct result of disrupted arteriovenous specification or whether EC fail to undergo proper arteriovenous specification as a by-product of enlarged, malformed vessels that result from aberrant responses to shear (such as failure to migrate against the direction of flow ^49, 55^) remains unclear.

Lastly, Notch is an important regulator of hemogenic endothelial cell development in the yolk sac and embryonic aorta–gonad–mesonephros region ^27, 56^, and circulating yolk sac-endothelium–derived hematopoietic progenitors have recently been shown to reintegrate into the developing vasculature ^9, 10^. Thus, it is interesting to speculate that Notch may contribute to arteriovenous network formation, in part, by regulating the relative abundance of these two endothelial cell sources, which may have different propensities for arterial versus venous identity. However, much more work is needed to address these possibilities.

### Ephrin/Eph

The ephrin family of transmembrane ligands and their cognate Eph receptors mediate cell–cell signaling between adjacent cells and often involve the repulsion of Eph receptor–expressing cells from ephrin-expressing neighbors. In a landmark study of the developing vasculature, Wang *et al*. ^11^ found that ephrinB2 expression is highly enriched in arteries and EphB4 expression is enriched in veins. Expression of both genes is observed prior to the onset of blood flow, suggesting that they participate in a genetic arteriovenous program. Furthermore, EphB4 venous expression depends upon arterial expression of ephrinB2 ^11^, suggesting that during development arterial specification may drive venous specification via ephrin-Eph signaling. Mutations that affect the *EphB4* gene or downstream Ras signaling are associated with the autosomal-dominant congenital vascular disease, capillary malformation-arteriovenous malformation (CM-AVM), wherein patients present with numerous cutaneous capillary malformations as well as AVMs ^57^.

### Transforming growth factor-beta

The TGF-β superfamily of soluble ligands and their cognate membrane-bound receptors play a variety of key roles during vessel development. This pathway includes signaling through the TGF-β1-TGFβR2-Alk5 ligand-receptor complex, which predominantly activates Smads2/3 signaling, as well as Bone morphogenetic protein (BMP) 9/10-Alk1-Eng ligand-receptor signaling, which predominantly activates Smads1/5/8 ^58^. Specifically, signaling through TGF-β1-TGFβR2 typically mediates mural cell recruitment and differentiation ^59^, whereas Alk1/Eng signaling regulates endothelial cell quiescence, limits vessel caliber, and enables arteriovenous specification ^54, 55, 60^. However, there is also evidence of significant cross-talk between distinct TGF-β signaling pathways ^61, 62^ as well as between TGF-β superfamily pathways and other cell signaling pathways (for example, Notch) and that it is the balance of Smad signaling activation via these distinct pathways that establishes proper vessel formation ^63, 64^. Indeed, patients bearing heterozygous mutations affecting either Alk1 or Eng, or downstream Smad4, exhibit the congenital disease hereditary hemorrhagic telangiectasia (HHT), which is characterized by microvascular overgrowth and the focal appearance of large-caliber AVMs that lack clear arterial or venous identity ^60, 65^.

Several recent studies have focused on the cross-talk between BMP and Notch signaling pathways to modulate endothelial cell behavior during vessel development. BMP-activated sprouting angiogenesis is negatively regulated by Notch upregulation of Smad6, an inhibitor of BMP signaling ^66^. Meanwhile, nuclear translocation of BMP-activated phospho-Smads not only upregulates BMP target genes but also participates in the Notch/RBPJ-κ gene regulatory complex to regulate Notch-activated transcriptional responses ^67^. Consequently, inhibited expression of endothelial-expressed BMP regulatory proteins, BMPER and TWSG1, disrupts Notch signaling and expression of arterial identity genes, resulting in increased venous specification in zebrafish embryos ^68^. Alk1 inhibition also depresses Notch signaling and produces AVMs in mice ^54^. In separate studies, BMPER is reported to activate ERK1/2 signaling ^69^ (which promotes arterial specification ^38^) while the Alk1 co-receptor Endoglin suppresses PI3K/Akt signaling (which promotes venous identity ^38^) to support endothelial cell migration against the direction of blood flow, a process hypothesized to support arteriogenesis and prevent AVMs ^70^. Thus, currently available evidence suggests that BMP9/10-Alk1 signaling may regulate arteriovenous specification, at least in part, by modulating endothelial cell responsiveness to VEGF- and Notch-activated signaling. However, other studies suggest that other endothelial cell behaviors, such as responsiveness to shear or migration (or both), are also affected ^49, 55^.

### Connexins

Membrane-expressed connexin (also known as Cx) proteins form multimeric complexes (termed connexons) that dock with connexons of adjacent cells to form intercellular channels (termed gap junction channels) that mediate passage of ions and small signaling molecules to support electrochemical coupling and intercellular communication. At least four connexin proteins (Cx37, Cx40, Cx43, and Cx45) are commonly reported at endothelial cell–cell junctions of the blood vasculature ^71, 72^, and some studies report endothelial expression of a fifth connexin (Cx32) ^73, 74^. Furthermore, an additional connexin (Cx47) is expressed in lymphatic endothelial cells and contributes to lymphatic vessel development ^75^. Of the commonly studied vascular connexins, Cx40 (encoded by the gene *Gja5*) is well recognized as a potent marker of arterial endothelial cells owing to its high expression in arteries invested with smooth muscle ^14, 17^. Deletion of this connexin inhibits flow-activated arterial specification in the chick ^14^ and affects sprouting angiogenesis and mural cell recruitment in the neonatal mouse retina ^76^. Furthermore, loss of Cx40 potentiates the appearance of AVMs in Alk1-haploinsufficient animals ^77^, suggesting that it may suppress the formation of these vascular defects in wild-type animals, at least in part, by functioning downstream of BMP9/10-Alk1 signaling. Recently, Su *et al*. ^26^ employed a single-cell transcriptomic analysis of developing coronary vessels to identify a Cx40-enriched population of venous-originating “pre-artery” cells that express markers of mature arteries. The majority of these cells were later found to line the coronary arteries and were excluded from coronary veins, suggesting that expression of Cx40 is a critical intermediary step for arterial identity acquisition.

Endothelial-expressed Cx37 is also almost exclusively expressed in large arteries of the adult vasculature ^78^ as well as in developing arteries and arterioles of remodeling vessels ^17^. However, unlike Cx40, Cx37 is additionally expressed in remodeling capillaries and in arteriolar vessels that have yet to be invested with mural cells ^17^, suggesting that it may play an earlier role than Cx40 in arteriovenous specification during development. Deletion of Cx37 disrupts developmental and injury-induced vessel growth and remodeling ^17, 79^, and transgenic ablation of both connexins in combination results in embryonic lethality due to failure of the vasculature to form ^80^, suggesting that Cx37 and Cx40 play essential and possibly distinct roles during vessel development. For example, many connexins regulate cell proliferation, and Cx37 is a particularly potent inhibitor of cell cycle progression ^81^. Cx37 directly modulates endothelial cell cycle status downstream of flow-activated Notch signaling by upregulating p27, causing late G _1_ arrest to enable expression of arterial genes Cx40 and ephrinB2 ^17^. It is possible that Cx37 plays a similar cell cycle arrest role to enable specification towards other endothelial cell fates. In support of this possibility, transgenic ablation of one or both copies of Cx37 is associated with endothelial cell hyperproliferation and defects in not only arterial development ^17^ but also venous ^82^ and lymphatic ^83, 84^ development.

### MicroRNAs

A growing body of evidence indicates that microRNA (miRNA) species likely play an important, if currently underappreciated, role in endothelial cell specification. Although several studies show that miRNAs are necessary for endothelial cell differentiation from angioblasts, less is known about their involvement in specifying endothelial cell phenotypes. Endothelial and mural cells express numerous miRNA species, and miRNA processing machinery, including *Drosha* and *Dicer*, appears to be crucial for vessel development ^85, 86^. Mutants lacking expression of *Dicer* in Etv2-positive mesodermal progenitor cells exhibit defects in vessel remodeling and patterning due, at least in part, to loss of miR-130a expression ^87^. Meanwhile, miR-27b is required for venous formation in zebrafish ^88^, and miR-181 destabilizes expression of Prox1, a key regulator of lymphatic endothelial cell specification and maintenance ^89^. In addition, endothelial-specific mutation of *Drosha* in mice produces leaky, dilated microvessels and aberrant arteriovenous connections (but lack clear AVMs), and missense point mutations in the *Drosha* gene are more prevalent among patients with HHT compared with healthy populations, suggesting that miRNA processing defects may contribute to the pathogenesis of this disease or modulate its severity or do both ^86^. Taken together, these studies suggest that miRNAs likely play a broad role in endothelial cell specification and vascular remodeling. However, more work is needed to identify critical miRNA regulators of these processes and to fully elucidate their molecular roles.

## Conclusions

Endothelial cell specification toward arterial and venous fates is critical for the formation and remodeling of the blood circulatory system during development and post-natally. Failure of the vasculature to properly undergo arteriovenous specification may contribute to the malformation or dysfunction of blood vessels, such as occurs in patients with HHT, who exhibit aberrant vessel structures that compromise quality of life and that can even be fatal.

**Figure 1.**
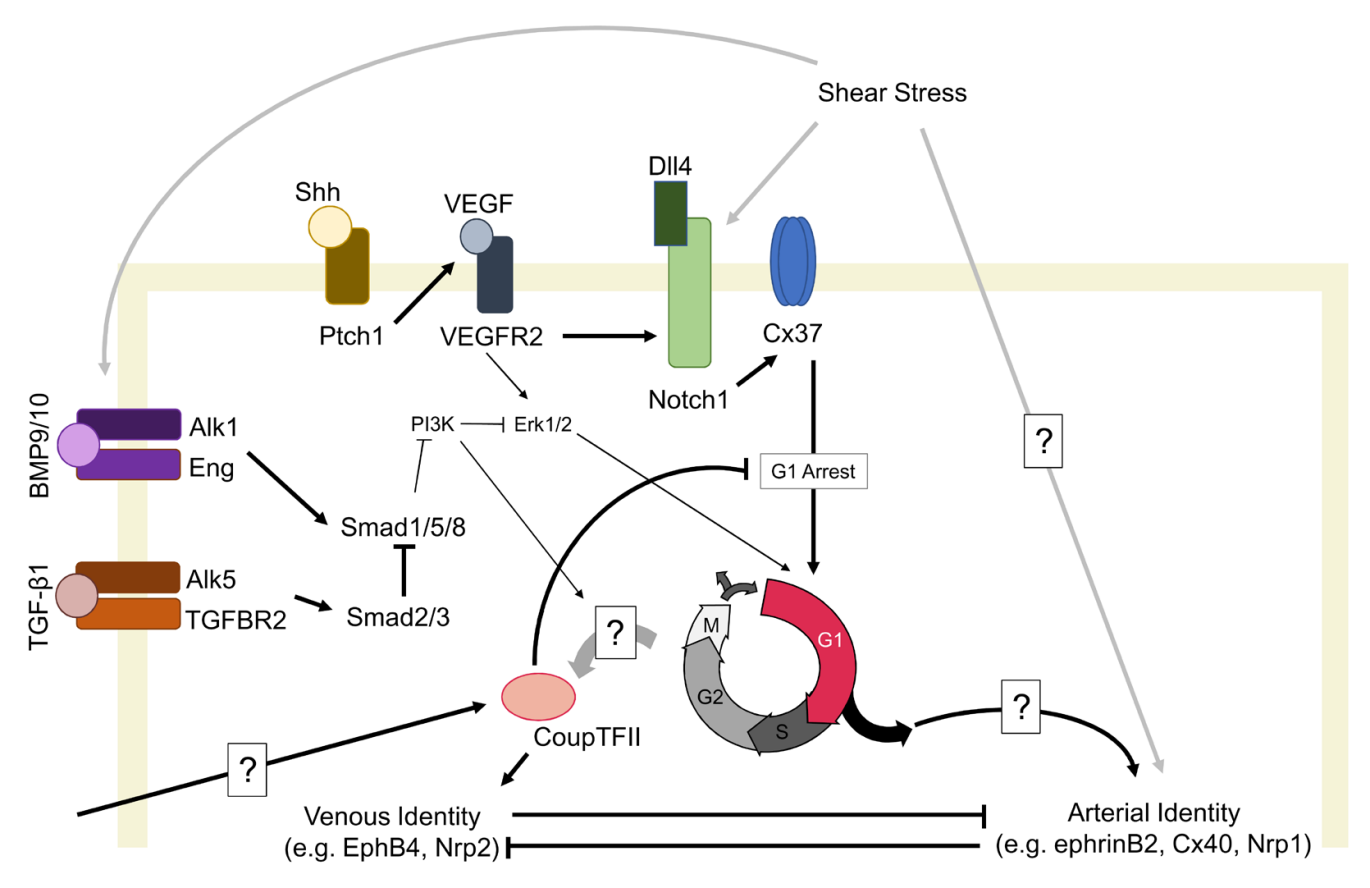
A proposed model of the regulation of arteriovenous specification in primitive endothelial cells, highlighting key players and some of the evidence of cell signaling cross-talk. A proposed model of the regulation of arteriovenous specification in primitive endothelial cells, highlighting key players and some of the evidence of cell signaling cross-talk.

During normal development, acquisition of arterial identity is driven by a molecular program (see proposed model, Figure 1) that includes Hedgehog, VEGF, Notch, and connexin signaling and downstream PI3K and MAPK signaling. This pathway is modulated by TGF-β signaling and miRNAs and is antagonized by COUP-TFII, which promotes venous formation. Although early endothelial specification events may occur via a “hardwired” genetic program prior to the onset of blood flow, hemodynamic flow is precisely calibrated to arteriovenous identity and vessel identity is sustained by blood flow forces. Recent studies suggest that flow-sensitive regulation of Notch signaling ^48^ may play a central role in modulating endothelial cell cycle state to enable the specification of different endothelial cell phenotypes in distinct cell cycle states ^17^; however, this requires further investigation.

An improved understanding of the molecular mechanisms that regulate the initial acquisition of endothelial cell identity, as well as the signals that sustain specialized vessel structures and functions, is therefore an important frontier for new and ongoing research in the field of vascular biology. Additional insights into the molecular regulation of arteriovenous specification will profoundly influence our understanding of the physiology of vessel maintenance and the pathophysiology of numerous diseases involving disorganized vessel growth and remodeling.

## Abbreviations

Akt, protein kinase B; Alk, activin receptor-like kinase; AVM, arteriovenous malformation; BMP, bone morphogenetic protein; COUP-TFII, COUP transcription factor 2; Cx, connexin; HHT, hereditary hemorrhagic telangiectasia; MAPK, mitogen-activated protein kinase; miRNA, microRNA; PI3K, phosphatidylinositol-3-kinase; Shh, Sonic Hedgehog; Smo, Smoothened; TGF-β, transforming growth factor-beta; VEGF, vascular endothelial growth factor; VEGFR2, vascular endothelial growth factor receptor type 2
